# Classification of Periapical and Bitewing Radiographs as Periodontally Healthy or Diseased by Deep Learning Algorithms

**DOI:** 10.7759/cureus.60550

**Published:** 2024-05-18

**Authors:** Muhammet Burak Yavuz, Nichal Sali, Sevda Kurt Bayrakdar, Cemre Ekşi, Büşra Seda İmamoğlu, İbrahim Şevki Bayrakdar, Özer Çelik, Kaan Orhan

**Affiliations:** 1 Periodontology, Faculty of Dentistry, Eskişehir Osmangazi University, Eskişehir, TUR; 2 Orthodontics, Hamidiye Faculty of Dental Medicine, University of Health Sciences, Istanbul, TUR; 3 Oral and Maxillofacial Radiology, Faculty of Dentistry, Eskişehir Osmangazi University, Eskişehir, TUR; 4 Mathematics and Computer Science, Faculty of Science, Eskisehir Osmangazi University, Eskişehir, TUR; 5 Oral, Dental, and Maxillofacial Radiology, Faculty of Dentistry, Ankara University, Ankara, TUR

**Keywords:** periodontal health, periodontal disease, periapical, bitewing, artificial intelligence

## Abstract

Objectives

The aim of this artificial intelligence (AI) study was to develop a deep learning algorithm capable of automatically classifying periapical and bitewing radiography images as either periodontally healthy or unhealthy and to assess the algorithm's diagnostic success.

Materials and methods

The sample of the study consisted of 1120 periapical radiographs (560 periodontally healthy, 560 periodontally unhealthy) and 1498 bitewing radiographs (749 periodontally healthy, 749 periodontally ill). From the main datasets of both radiography types, three sub-datasets were randomly created: a training set (80%), a validation set (10%), and a test set (10%). Using these sub-datasets, a deep learning algorithm was developed with the YOLOv8-cls model (Ultralytics, Los Angeles, California, United States) and trained over 300 epochs. The success of the developed algorithm was evaluated using the confusion matrix method.

Results

The AI algorithm achieved classification accuracies of 75% or higher for both radiograph types. For bitewing radiographs, the sensitivity, specificity, precision, accuracy, and F1 score values were 0.8243, 0.7162, 0.7439, 0.7703, and 0.7821, respectively. For periapical radiographs, the sensitivity, specificity, precision, accuracy, and F1 score were 0.7500, 0.7500, 0.7500, 0.7500, and 0.7500, respectively.

Conclusion

The AI models developed in this study demonstrated considerable success in classifying periodontal disease. Future applications may involve employing AI algorithms for assessing periodontal status across various types of radiography images and for automated disease detection.

## Introduction

Periodontitis is a chronic inflammatory condition that impacts the supportive tissues around the teeth, leading to symptoms like periodontal pockets and irreversible attachment loss, potentially resulting in tooth loss [1]. Given its high prevalence, detecting periodontitis relies on various clinical parameters and radiographic imaging [2]. While both intraoral and extraoral radiographs are valuable for detection, bitewing, periapical, and panoramic radiographs are commonly employed methods [3]. The 2017 periodontal disease classification recommends assessing alveolar bone loss through a comparison of a patient's five-year radiographs as evidence of rapid progression of periodontitis [4].

Periapical radiography defines intraoral techniques designed to depict teeth and the surrounding tissues around the apex. Each image typically depicts two to four teeth, providing detailed information about the teeth and the surrounding alveolar bone [5]. Bitewing radiographs derive their name from the original technique that required the patient to bite down on a small wing attached to the mouthpiece film packet. The acquired radiographs are used to gain information about the crowns of the premolars and molars on one side of the jaws and the marginal periodontal tissues [6]. While bitewing radiographs are commonly used in dentistry for detecting decay, they have also been shown to be useful in identifying periodontal disease [7,8].

The term artificial intelligence (AI) refers to the capability of machines to perform tasks traditionally done by humans [9]. In dentistry and medicine, convolutional neural networks (CNNs), a type of artificial neural network, are employed for interpreting images and diagnosing diseases based on radiographs. Numerous studies have explored CNN's utility in areas such as detecting periodontal disease [10], identifying cavities [11], spotting periapical lesions [12], and measuring alveolar bone [13] using dental radiographs. These studies demonstrate that CNN systems can aid healthcare providers in diagnosing dental conditions. By integrating CNN systems into the diagnostic phase, they can serve as an alert mechanism for clinicians, helping identify periodontal diseases that might otherwise go unnoticed due to factors such as oversight, lack of expertise, or fatigue. In light of this information, the present study aims to utilize CNN technology to classify periapical and bitewing images, commonly utilized in dental practice, as either periodontally healthy or indicative of periodontitis.

## Materials and methods

Study design and ethical approval

This retrospective study was conducted using a dataset comprising of anonymized periapical and bitewing images sourced from the Department of Oral and Maxillofacial Radiology at Eskişehir Osmangazi University's Faculty of Dentistry, Eskişehir, Turkey. Approval for the study was granted by the Non-Interventional Clinical Research Ethics Committee (decision date and number: 08.07.2019/2019-227), and the study protocol adhered to the ethical guidelines outlined in the Declaration of Helsinki.

Patient selection and imaging

Periapical and bitewing images with artifacts or partial/severe distortion were excluded from the dataset, ensuring each image was used only once in the study. Furthermore, all radiographic images employed in this research were acquired using the same device (ProX, Planmeca, Helsinki, Finland), utilizing irradiation parameters of 60 kV, 7 mA, and 0.08 s. An experienced periodontologist (NS, with a minimum of five years of professional experience) initially evaluated the radiographic images, categorizing them as either periodontally healthy or unhealthy. Subsequently, the classified images underwent re-evaluation and scrutiny by one radiologist and three periodontologists (İŞB, SKB, MBY, CE). It was found that the observer who evaluated the radiographs showed high consistency in diagnosing lack of health/health at different times. When repeated evaluations were compared, kappa values were in excellent agreement for both radiograph types (K = 0.81-0.99). Radiographs lacking consensus were excluded from the study. The final dataset comprised 1120 periapical radiographs and 1498 bitewing radiographs, with a total of 560 periodontally healthy and 560 periodontally ill periapical images, as well as 749 periodontally healthy and 749 periodontally ill bitewing images.

The presence or absence of resorption in the alveolar bone crest was assessed based on the distance between the cementum-enamel junction of the teeth and the alveolar crest. Radiographs demonstrating no loss of the alveolar bone apex and devoid of any bone defects were categorized into the periodontally healthy group. Conversely, radiographs with a distance of 2 mm or more between the alveolar bone crest and the cementum-enamel junction were classified into the periodontally ill group [14].

Before training, preprocessing steps were implemented, including resizing the images to dimensions of 640 × 640 pixels. The dataset was then partitioned into three distinct groups: training, validation, and test sets for both periapical and bitewing images (Table 1). In the periapical image dataset, the training set comprised 896 images (80%), the validation set contained 112 images (10%), and the test set included 112 images (10%). For bitewing radiographs, 1202 images (80%) were allocated to the training set, 148 images (10%) to the validation set, and 148 images (10%) to the test set.

**Table 1:** Bitewing-periapical dataset (n = radiograph numbers)

Bitewing-periapical dataset	Healthy case (n)	Unhealthy case (n)	Total
Bitewing training	601	601	1202
Bitewing validation	74	74	148
Bitewing testing	74	74	148
Periapical training	448	448	896
Periapical validation	56	56	112
Periapical testing	56	56	112

An AI model was created to classify periapical and bitewing images as either periodontally healthy or unhealthy, employing 300 epochs with the You Only Look Only Once classification model (YOLOv8-cls, Ultralytics, Los Angeles, California, United States). YOLOv8-cls was selected due to its advanced object classification architecture, offering heightened sensitivity and superior performance in terms of accuracy and speed compared to the YOLOv8 system. Throughout the model development phase, the PyTorch library (v. 3.6.1; Python Software Foundation, Wilmington, Delaware, United States) was utilized, operating on a computer equipped with 16 GB RAM and an NVIDIA GeForce GTX 1660 TI graphics card (NVIDIA Corporation, Santa Clara, California, United States). The model's classification success was assessed using the confusion matrix method, analyzing the test dataset.

Statistical analysis

Statistical analyses were conducted utilizing a confusion matrix, a valuable table that summarizes the predicted and actual states. Sensitivity, specificity, precision, accuracy, and F1 score were calculated with the confusion matrix. Then by these calculation methods (sensitivity: TPR = TP / (TP + FN) (TPR: true positive rate, TP: true positive, FN: false negative), specificity: SPC = TN / (FP + TN) (SPC: specificity, TN: true negative, FP: false positive), precision: PPV = TP / (TP + FP) (PPV: positive predictive value), accuracy: ACC = (TP + TN) / (P + N) (accuracy: ACC, P: positive, N: negative) and F1 Score: F1 = 2TP / (2TP + FP + FN)), sensitivity, specificity, precision, accuracy, and F1 score were determined.

## Results

Results for bitewing radiographs

**Figure 1:**
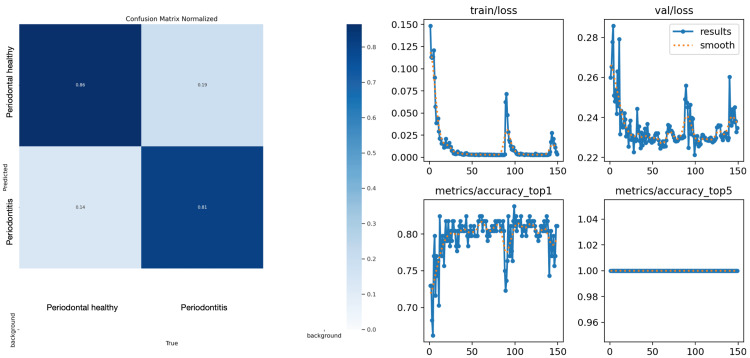
Charts depicting the performance evaluation results of the artificial intelligence (AI) algorithm developed for bitewing radiographs In the graphics, the X-axis shows the epoch number, and the Y-axis shows train/loss, validation loss, accuracy top1, and accuracy top5.

The analysis of the success of the AI system developed for bitewing radiographs revealed that the sensitivity, specificity, precision, accuracy, and F1 score were 0.8243, 0.7162, 0.7439, 0.7703, and 0.7821, respectively. Specifically, the algorithm correctly identified 61 out of 74 periodontally healthy images in the test group as healthy, and 53 out of 74 periodontally unhealthy images as unhealthy. The results of the system's performance are depicted in Tables 2, 3, as well as Figure 1.

**Table 2:** Performance of the developed YOLOv8-cls* algorithms in periodontal condition classification (n = radiograph number) *Ultralytics, Los Angeles, California, United States

	Bitewing		Periapical	
Class	Periodontally healthy (n)	Periodontally unhealthy (n)	Periodontally healthy (n)	Periodontally unhealthy (n)
Periodontally healthy (n)	61	21	42	14
Periodontally unhealthy (n)	13	53	14	42

**Table 3:** Evaluation results of artificial intelligence (AI) performance with a confusion matrix TPR, true positive rate; TP, true positive; FN, false negative; SPC, specificity; TN, true negative; FP, false positive; PPV, positive predictive value; ACC, accuracy; P, positive; N, negative

Measure	Value-bitewing	Value-periapical	Derivations
Sensitivity	0.8243	0.7500	TPR = TP / (TP + FN)
Specificity	0.7162	0.7500	SPC = TN / (FP + TN)
Precision	0.7439	0.7500	PPV = TP / (TP + FP)
Accuracy	0.7703	0.7500	ACC = (TP + TN) / (P + N)
F1 score	0.7821	0.7500	F1 = 2TP / (2TP + FP + FN)

Results for periapical radiographs

**Figure 2:**
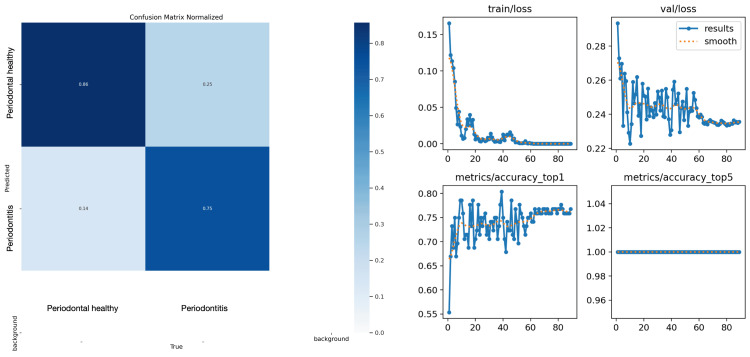
Charts depicting the performance evaluation results of the artificial intelligence (AI) algorithm developed for periapical radiographs In the graphics, the X-axis shows the epoch number, and the Y-axis shows train/loss, validation loss, accuracy top1, and accuracy top5.

When assessing the performance of the AI system developed for periapical radiographs, the sensitivity, specificity, precision, accuracy, and F1 score were found to be 0.7500 each. Specifically, the algorithm correctly identified 42 out of 56 periodontally healthy images in the test group as healthy, and 42 out of 56 periodontally unhealthy images as unhealthy. The results of the system's performance are presented in Tables 2, 3, as well as Figure 2.

**Figure 3:**
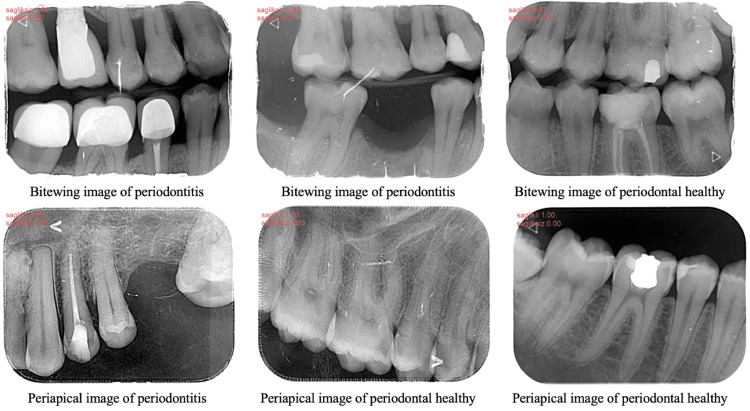
All images presented showing the output of the artificial intelligence (AI) algorithm developed for bitewing and periapical radiographs indicate that the system correctly diagnosed all images

A few of the bitewing and periapical images correctly diagnosed by the AI algorithm are shown in Figure 3.

## Discussion

Despite their inability to directly visualize periodontal pockets and gingivitis, periapical and bitewing radiographs have demonstrated efficacy in detecting alveolar bone and osseous lesions supporting the teeth [15]. Bitewing radiographs are particularly valuable for monitoring crestal bone height in posterior teeth alongside caries detection, whereas periapical radiographs offer insights into potential loss of bone support using the long cone paralleling technique [16]. Leveraging this knowledge, the current study employed a CNN system to diagnose periodontal health and disease from periapical and bitewing images.

AI systems hold promise as supportive tools in decision-making processes for dental diagnosis, particularly through the interpretation of dental radiographs. They can guide inexperienced dentists and dental students, as well as alleviate diagnostic burdens on specialist physicians and experienced dentists [17]. Full-mouth radiographs remain standard in dentistry due to their minimal radiation exposure and detailed periodontal assessment [18,19]. The utilization of CNN-based AI systems for storing and analyzing these radiographic images actively used in dentistry may play a role in early diagnosis, reducing the workload of dentists and mitigating the inadequacy of diagnosis attributable to dentists. In line with current insights, the primary objective of this study was to automatically assess intraoral radiographs, commonly used in dental settings, utilizing a developed AI algorithm.

Upon reviewing the literature, while numerous studies exist in this domain, to our knowledge, this is the first academic study to assess an algorithm capable of automatically detecting periodontal status from both bitewing and periapical radiographs. For example, previous studies have explored various applications of CNN-based AI systems in dentistry, such as detecting alveolar bone loss via panoramic radiography [17,20], measuring alveolar bone loss using periapical radiographs in patients with periodontal disease [21], and diagnosing caries, apical lesions, and periodontitis through periapical radiographs [22]. Additionally, algorithms have been developed to assess the number of existing teeth using panoramic, periapical, and bitewing images [23], to evaluate marginal bone loss around implants [24], and to detect periodontal inflammation, tooth numbering, gingival overgrowth, and frenulum connection through intraoral photographs [25,26]. The results of these studies are promising for the potential integration of AI systems into the field of dentistry in the future.

In their study aimed at detecting periodontal bone loss using 2001 panoramic images, Krois et al. [20] reported mean sensitivity and specificity values of 0.81 each for CNN-based AI results. Comparing the results of this study, the AI outputs were contrasted with those of six dentists, revealing a higher accuracy rate for the AI system. While the dentists exhibited a mean accuracy of 0.76, the CNN-based system achieved a mean accuracy of 0.81. Although these results may not reach statistical significance, they suggest that AI systems have the potential to streamline the diagnostic process for dentists in identifying periodontal disease.

In their study designed to quantify the amount of alveolar bone loss through periapical radiographs, Lin et al. [21] assessed bone loss across 18 periapical images. They highlighted the potential for early detection of alveolar bone loss and expedited diagnosis of periodontitis if implemented in clinical practice, allowing for a margin of deviation of up to 25%. The approaches employed by Krois et al. and Lin et al. differ significantly from the current study, as they did not involve classification methods.

In another study focusing on detecting bone loss around implants, Liu et al. [24] utilized a regions-with-CNN (R-CNN)-based model to analyze 1670 periapical images, divided into 1370 training, 150 validation, and 150 test sets. They compared the model's ability to detect marginal bone loss around implants with that of assistant dentists and specialist dentists using kappa (κ) statistics. The study results indicated that while the success of the AI model was lower than that of the specialist dentist, it was on par with that of the assistant dentist. Although not evaluated in the current study, future research should explore the performance of AI systems developed in comparison to physicians with varying levels of experience. This could provide a more comprehensive demonstration of the system's success.

In a separate study by Chen et al. [22], based on R-CNN, they classified caries, periodontitis, and apical periodontitis into mild, moderate, and severe categories using 2900 periapical radiographs. They aimed to assess the success of R-CNN based on disease severity. Their results revealed that R-CNN exhibited greater success in detecting severe caries and apical periodontitis compared to low-level caries.

Lee et al. [10] investigated 1740 periapical films using the CNN algorithm. They reported an accuracy rate of 81% for diagnosing periodontitis in premolar teeth and 76.7% for molar teeth. Additionally, the study found that CNN accurately predicted teeth requiring extraction, with a success rate of 82.8% for premolars and 73.4% for molars. Furthermore, another study demonstrated that AI algorithms can automatically determine not only bone loss but also stages of periodontitis according to the latest classification of periodontal diseases [13].

In a study by Khan et al., an AI system was developed for assessing bone losses and furcation defects on periapical radiographs, demonstrating successful outcomes [27]. More recently, Kurt-Bayrakdar et al. reported the successful detection of vertical, horizontal, and furcation defects using an AI algorithm developed with U-NET architecture (Albert Ludwig University of Freiburg, Freiburg im Breisgau, Baden-Württemberg, Germany) on 1121 panoramic radiographs [28]. A study very similar to the current study was conducted by Kurt-Bayrakdar et al. in 2020 [17]. In this study, an AI system was developed utilizing 2276 panoramic radiography images and Google Net Inception-v3 architecture (Google LLC, Mountain View, California, United States). The system's performance was presented, showcasing its ability to distinguish panoramic radiographs as periodontally diseased or healthy, with reported success rates exceeding 90%. The key similarity between these studies lies in their focus on developing models for classifying periodontal status using a classification approach, rather than utilizing detection or segmentation methods for identifying pathology. In other words, while different radiographs and algorithms were employed, the material and methods sections of the studies exhibit notable similarities. The study by Kurt-Bayrakdar et al. indeed achieved higher success rates, which could be attributed to the utilization of a larger dataset [17]. 

The study results uncovered that the algorithm developed for bitewing radiographs was more successful in evaluating periodontal status compared to those developed for periapical radiographs. The observed superior performance of the algorithm developed for bitewing radiographs compared to the ones developed for periapical radiographs may be attributed to the larger dataset used for bitewing radiographs. This aligns with the notion that success tends to increase with larger training datasets in AI training, as suggested in the literature [29]. Another factor influencing this result could be the lack of standardization in the angles of periapical radiographs. Hence, radiographs taken using a parallel technique for periodontal diagnosis, which are deemed more reliable, have been recommended [30]. Although the present study did not utilize radiography images acquired with the parallel technique, future studies could potentially yield more successful results by adhering to this recommendation.

It can be asserted that the major limitation of the study lies in the utilization of a restricted number of periapical and bitewing radiographic images obtained from a single center. Additionally, the development of an algorithm capable of detecting bone defects, rather than solely discerning disease/health status, could offer greater depth to the periodontology literature by providing more detailed information. Another limitation is the reliance on a single AI architecture in the study. A more comprehensive analysis could have been achieved by comparing the efficacy of algorithms developed using various AI architectures.

## Conclusions

Our study underscores the potential of AI to automatically identify periodontal pathologies that might otherwise go unnoticed. This capability has the potential to reduce radiation exposure by minimizing the need for repeated evaluations, halt the silent progression of periodontal disease, and facilitate earlier treatment initiation. It is evident that the integration of AI systems into radiographic diagnosis will streamline the workflow of healthcare providers. The results of the present study emphasize the necessity for larger radiological datasets to achieve more accurate periodontal status determination (unhealthy/healthy) using these systems. In conclusion, despite its limitations, this study corroborates the notion that AI can assist dentists in diagnosing and treating diseases earlier and with greater precision, consistent with previous studies in the literature.
